# Comparative effectiveness of intracranial hypertension management guided by ventricular versus intraparenchymal pressure monitoring: a CENTER-TBI study

**DOI:** 10.1007/s00701-022-05257-z

**Published:** 2022-06-01

**Authors:** Victor Volovici, Dana Pisică, Benjamin Y. Gravesteijn, Clemens M. F. Dirven, Ewout W. Steyerberg, Ari Ercole, Nino Stocchetti, David Nelson, David K. Menon, Giuseppe Citerio, Mathieu van der Jagt, Andrew I. R. Maas, Iain K. Haitsma, Hester F. Lingsma, on behalf of CENTER-TBI investigators, participants for the ICU stratum, Cecilia Åkerlund, Krisztina Amrein, Nada Andelic, Lasse Andreassen, Gérard Audibert, Philippe Azouvi, Maria Luisa Azzolini, Ronald Bartels, Ronny Beer, Bo-Michael Bellander, Habib Benali, Maurizio Berardino, Luigi Beretta, Erta Beqiri, Morten Blaabjerg, Stine Borgen Lund, Camilla Brorsson, Andras Buki, Manuel Cabeleira, Alessio Caccioppola, Emiliana Calappi, Maria Rosa Calvi, Peter Cameron, Guillermo Carbayo Lozano, Ana M. Castaño-León, Simona Cavallo, Giorgio Chevallard, Arturo Chieregato, Mark Coburn, Jonathan Coles, Jamie D. Cooper, Marta Correia, Endre Czeiter, Marek Czosnyka, Claire Dahyot-Fizelier, Paul Dark, Véronique De Keyser, Vincent Degos, Francesco Della Corte, Hugo den Boogert, Bart Depreitere, Dula Dilvesi, Abhishek Dixit, Jens Dreier, Guy-Loup Dulière, Erzsébet Ezer, Martin Fabricius, Kelly Foks, Shirin Frisvold, Alex Furmanov, Damien Galanaud, Dashiell Gantner, Alexandre Ghuysen, Lelde Giga, Jagos Golubovic, Pedro A. Gomez, Francesca Grossi, Deepak Gupta, Iain Haitsma, Eirik Helseth, Peter J. Hutchinson, Stefan Jankowski, Faye Johnson, Mladen Karan, Angelos G. Kolias, Daniel Kondziella, Evgenios Koraropoulos, Lars-Owe Koskinen, Noémi Kovács, Ana Kowark, Alfonso Lagares, Steven Laureys, Didier Ledoux, Aurelie Lejeune, Roger Lightfoot, Alex Manara, Costanza Martino, Hugues Maréchal, Julia Mattern, Catherine McMahon, Tomas Menovsky, Benoit Misset, Visakh Muraleedharan, Lynnette Murray, Ancuta Negru, Virginia Newcombe, József Nyirádi, Fabrizio Ortolano, Jean-François Payen, Vincent Perlbarg, Paolo Persona, Anna Piippo-Karjalainen, Horia Ples, Inigo Pomposo, Jussi P. Posti, Louis Puybasset, Andreea Radoi, Arminas Ragauskas, Rahul Raj, Jonathan Rhodes, Sophie Richter, Saulius Rocka, Cecilie Roe, Olav Roise, Jeffrey V. Rosenfeld, Christina Rosenlund, Guy Rosenthal, Rolf Rossaint, Sandra Rossi, Juan Sahuquillo, Oddrun Sandrød, Oliver Sakowitz, Renan Sanchez-Porras, Kari Schirmer-Mikalsen, Rico Frederik Schou, Peter Smielewski, Abayomi Sorinola, Emmanuel Stamatakis, Nina Sundström, Riikka Takala, Viktória Tamás, Tomas Tamosuitis, Olli Tenovuo, Matt Thomas, Dick Tibboel, Christos Tolias, Tony Trapani, Cristina Maria Tudora, Peter Vajkoczy, Shirley Vallance, Egils Valeinis, Zoltán Vámos, Gregory Van der Steen, Roel P. J. van Wijk, Alessia Vargiolu, Emmanuel Vega, Anne Vik, Rimantas Vilcinis, Petar Vulekovic, Guy Williams, Stefan Winzeck, Stefan Wolf, Alexander Younsi, Frederick A. Zeiler, Agate Ziverte, Hans Clusmann, Daphne Voormolen, Jeroen T. J. M. van Dijck, Thomas A. van Essen

**Affiliations:** 1 Department of Neurosurgery, Erasmus MC, Rotterdam, The Netherlands; 2 Department of Public Health, Center for Medical Decision Making, Erasmus MC University Medical Center, Erasmus MC Stroke Center, Doctor Molewaterplein 40, 3015 GD Rotterdam, The Netherlands; 3 Department of Medical Statistics and Bioinformatics, Leiden University Medical Center, Leiden, The Netherlands; 4 Division of Anesthesia, University of Cambridge, Addenbrooke’s Hospital, Cambridge, UK; 5 Neuroscience Intensive Care Unit, Department of Anesthesia and Critical Care, Fondazione IRCCS Ca’ Granda - Ospedale Maggiore Policlinico, Milan, Italy; 6 Department of Pathophysiology and Transplantation, University of Milan, Milan, Italy; 7 Section of Perioperative Medicine and Intensive Care, Department of Physiology and Pharmacology, Karolinska Institutet, Stockholm, Sweden; 8 School of Medicine and Surgery, University of Milan-Bicocca, Milan, Italy; 9 Neurointensive Care, San Gerardo Hospital, ASST, Monza, Italy; 10 Department of Intensive Care Adults, Erasmus MC, Rotterdam, The Netherlands; 11 Department of Neurosurgery, UZ Antwerp University Hospital, Edegem, Belgium

**Keywords:** External ventricular devices, Intraparenchymal monitors, Intracranial pressure monitoring, Severe TBI, Traumatic brain injury, CENTER-TBI, Intracranial hypertension, EVD, ICP

## Abstract

**Objective:**

To compare outcomes between patients with primary external ventricular device (EVD)–driven treatment of intracranial hypertension and those with primary intraparenchymal monitor (IP)–driven treatment.

**Methods:**

The CENTER-TBI study is a prospective, multicenter, longitudinal observational cohort study that enrolled patients of all TBI severities from 62 participating centers (mainly level I trauma centers) across Europe between 2015 and 2017. Functional outcome was assessed at 6 months and a year. We used multivariable adjusted instrumental variable (IV) analysis with “center” as instrument and logistic regression with covariate adjustment to determine the effect estimate of EVD on 6-month functional outcome.

**Results:**

A total of 878 patients of all TBI severities with an indication for intracranial pressure (ICP) monitoring were included in the present study, of whom 739 (84%) patients had an IP monitor and 139 (16%) an EVD. Patients included were predominantly male (74% in the IP monitor and 76% in the EVD group), with a median age of 46 years in the IP group and 48 in the EVD group. Six-month GOS-E was similar between IP and EVD patients (adjusted odds ratio (aOR) and 95% confidence interval [CI] OR 0.74 and 95% CI [0.36–1.52], adjusted IV analysis). The length of intensive care unit stay was greater in the EVD group than in the IP group (adjusted rate ratio [95% CI] 1.70 [1.34–2.12], IV analysis). One hundred eighty-seven of the 739 patients in the IP group (25%) required an EVD due to refractory ICPs.

**Conclusion:**

We found no major differences in outcomes of patients with TBI when comparing EVD-guided and IP monitor–guided ICP management. In our cohort, a quarter of patients that initially received an IP monitor required an EVD later for ICP control. The prevalence of complications was higher in the EVD group.

**Protocol:**

The core study is registered with ClinicalTrials.gov, number NCT02210221, and the Resource Identification Portal (RRID: SCR_015582).

**Supplementary Information:**

The online version contains supplementary material available at 10.1007/s00701-022-05257-z.

## Introduction

In severe traumatic brain injury (TBI), intracranial pressure (ICP) is frequently monitored to guide treatment of intracranial hypertension [5].

Two main groups of devices are used to monitor ICP [21]. Intraparenchymal (IP) monitors are usually inserted in the intensive care unit (ICU) or the operating room (OR) by drilling a hole in the skull, piercing the meninges and inserting the thin catheter in the brain parenchyma of the right frontal region (or tailored to the expected maximum swelling area). External ventricular devices (EVDs) are usually inserted in the OR by drilling a larger burr hole above Kocher’s point and inserting the catheter in the lateral ventricles.

There is considerable practice variation with respect to the choice of monitoring device [5]. From a pathophysiological perspective, the use of an EVD instead of an IP monitor would offer more ICP control and therefore result in a better outcome [21]. This is because using an EVD enables not only intracranial pressure monitoring, but also ICP-lowering therapy: drainage of cerebrospinal fluid (CSF) that may obviate the need for other ICP lowering treatments, including decompressive craniectomy (DC). One of the major drawbacks of EVD use is a higher risk of complications, notably drain-related infections, compared to parenchymal monitors [21]. Furthermore, EVDs may lead to slit ventricles, and increase the risk of surgical complications such as hematoma formation.

A meta-analysis of our group which pooled the results of all available studies until 2018 [3, 21] showed no benefit in terms of mortality or functional outcome when using EVD instead of IP monitors. This meta-analysis overturned the result of the only randomized controlled trial (RCT) on the topic, which showed superiority of EVDs in terms of mortality and functional outcome [11]. A more recent retrospective analysis showed worse outcomes in patients treated with a primary EVD [3]. Explanations of these contradictory findings could be confounding by indication present in the retrospective observational studies, albeit adjusted with state-of-the-art statistical methods [1, 3, 10], and the limited generalizability of the RCT [11]. Historically, treatment guidelines for TBI indicated EVDs as “third tier” therapies [4]. In the most recent edition of the guidelines, their place in the severe treatment strategy is no longer stated. The only current recommendation is that CSF should be drained continuously.

Studying an isolated intervention is difficult in TBI patients, because of the strong interdependence of individual treatment modalities aimed at lowering intracranial hypertension, as well as the heterogeneity of the patient population [23]. This sometimes leads to confusing guideline recommendations because of the difficulty in generating robust evidence in TBI [22]. However, this variation does provide opportunities for comparative effectiveness research (CER) [12, 17, 24]. CER exploits practice variation by taking advantage of the “natural experiment” that occurs when patients go to different hospitals, each with their own treatment preferences. Analyzing the effect of treatment preference instead of actual treatment a patient received minimizes confounding by indication. Therefore, the treatment effect estimate from this analysis should have a lower risk of bias [7, 13].

Within a large prospective observational study, CENTER-TBI, we aimed to compare outcomes between patients with an EVD and patients with an IP monitor as the primary ICP monitoring modality. We hypothesized that patients receiving an EVD would have a better outcome due to the option to drain CSF, decreasing the need for third tier therapies and decompressive craniectomies.

## Methods

### Patient population

The CENTER-TBI study is a prospective, multicenter, longitudinal observational cohort study that enrolled patients of all TBI severities. TBI patients presenting within 24 h after injury with a clinical indication for a brain CT scan to one of the 62 participating study sites in Europe (mainly level 1 trauma centers), or referred from another hospital to the participating study site, were eligible for this study. Extensive details and the study design are available in a previous publication [14, 19]. For this study we included patients of all TBI severities admitted to the intensive care unit (ICU) with an indication for ICP monitoring.

### Patient characteristics

Baseline characteristics extracted from the CENTER-TBI database were age, sex, total injury severity score, pupillary reactivity, the most reliable Glasgow Coma Scale (GCS) and motor GCS score, pupillary reactivity, and injury cause. Furthermore, from the first CT scan, the following features were extracted: the presence of a skull fracture, epidural or subdural hematoma or traumatic subarachnoid hemorrhage, intraventricular hemorrhage, compression of basal cisterns, and midline shift.

### Outcomes and aims

The primary aims of this study were to assess 6-month mortality and unfavorable functional outcome in the EVD group compared to the IP monitor group. The co-primary outcomes were, therefore, mortality at 6 months and unfavorable functional outcome, as defined by the 6-month Glasgow Outcome Scale-Extended (GOS-E) of 4 or less (Supplementary Table 1).

The secondary outcomes were as follows: (a) the median daily therapy intensity level (TIL) for the first 12 days in the ICU together with the median ICP per day; the TIL is a validated measure of how much therapy a patient requires in order to control ICP; (b) hospital and ICU length of stay (LOS); (c) the use of secondary DC for refractory intracranial hypertension; (d) the use of other third tier therapies (barbiturate coma, hypothermia); (e) the risk of overall complications, defined as infection (meningitis/ventriculitis) and delayed hematoma (new intracerebral hematoma on follow-up radiological studies) or monitoring device malfunction; (f) mortality in the ICU and in the hospital; (g) the prevalence of cross-over (i.e., IP monitor patients eventually receiving an EVD); (h) ratio of time points with ICP above 20 mmHg and above 25 mmHg out of all recorded ICP time points. ICP was registered hourly during ICU stay for the first 10 days, and on days 12, 14, and 21. We only included “secondary” DCs, excluding DCs performed before insertion of the monitoring device, for a primary space-occupying lesion or for signs of intracranial hypertension on the first CT scan.

### Sensitivity analyses

The main analysis mirrored an “intention-to-treat” analysis from a trial. We defined the groups based on the first monitor received. Therefore, the entire subgroup of patients that received an EVD later on due to refractory hypertension was considered part of the IP monitor group even though they harbored both monitors.

As a sensitivity analysis, we analyzed all outcomes “as-treated,” by defining groups as “IP monitor only” and “EVD at any timepoint.”

As a second sensitivity analysis, we re-ran all analyses with 12-month mortality and unfavorable outcome as co-primary outcomes.

A final sensitivity analysis was the “complete case” analysis, which included only patients without missing data.

### Data analysis

Baseline characteristics were compared between patients who received an EVD or IP monitor with Student’s *t*-tests, Mann–Whitney *U* tests, chi-squared tests, or Fisher’s exact test. Furthermore, to provide a summary measure of “baseline risk” for each group, the probabilities of 6-month mortality and unfavorable outcomes were predicted based on baseline characteristics and the base IMPACT model [16] using logistic regression. The median predicted probability per treatment group was calculated, with the interquartile range (IQR).

Because we assume missing at random (MAR), we performed multiple imputation on the data to obtain 5 datasets. Outcomes were included in the imputation model (less than 15% missing).

To analyze which patient characteristics affected the choice for an EVD or IP monitor, logistic regression analysis was performed, with IP monitor as the reference outcome category. All relevant predictors from the descriptive analysis were included, but only those with a *p*-value below 0.2 in the full model were retained in the final model. To take center effects into account, the model was extended with a nested random intercept for center. For both models, the Nagelkerke *R*^2^ was calculated, to assess the proportion of variance in treatments that was explained by the different models.

The effects of EVD versus IP on outcome were estimated based on two methods.

First, an instrumental variable approach was used, with center preference for EVD over IP as instrument, both unadjusted and adjusted for confounders.

Second, we performed multivariable adjustment using variables that differed between the groups at baseline. To assess the effect of EVD versus IP on unfavorable outcome (GOS-E < 5), mortality, and decompressive craniectomy, logistic regression analysis was used. For the effect on length of hospital or ICU stay, a quasi-Poisson regression analysis was performed.

The adjusted IV analysis was considered the main analysis and these effect size estimates were reported. This choice was motivated by the considerable effect of adding a random intercept for center to the analysis of factors associated with choice of monitor. Using adjusted IV analysis minimized confounding by indication present in non-randomized data. Regarding outcomes for which the number of events was too small to use IV analysis (use of third tier therapies, complications, ratio of time points with ICP above 20 mmHg and above 25 mmHg out of all recorded ICP time points), we consider the main effect size estimate the one provided by multivariable confounder adjustment.

Instrumental variable (IV) analysis [7, 13] can theoretically correct for observed and unobserved confounders. To be valid, three assumptions should be met [13]. The relevance assumption was confirmed by logistic regression analysis. Most centers included in the study were level I trauma centers actively involved in TBI research with considerable experience in monitoring modalities, we therefore considered the exclusion assumption met. Furthermore, we verified this assumption in a previously published paper by our group [9]. For the exchangeability assumption, we considered previous research showing that in TBI, correlation between known confounders and center is low [6]. Given these arguments, we judged our instrument (center) for the IV analysis as being of moderate strength [15]. Clinical centers are an accepted and valid instrument in the scientific literature [6]. Centers including less than 10 patients were excluded. Our group’s previous research shows that a cut-off of 10 patients is a valid choice [7].

## Results

A total of 2138 patients were admitted to the ICU in the CENTER-TBI cohort, with median age of 49 years; 36% of whom were mild TBI (GCS 13–15). A total of 878 ICU patients were included in the present study. Of these, 739 (84%) patients received an IP monitor and 139 (16%) patients received an EVD (Table 1). For the main analysis (“intention-to-treat”), we included in the “IP monitor” group all patients who received an IP monitor first, including 187 who received an EVD for CSF drainage at later stages in their ICP management course. The instrumental variable analysis, which excluded centers with less than 10 patients, included 639 patients with an IP monitor and 115 with an EVD (Supplementary Table 2, Supplementary Fig. 1).

**Table 1:** Baseline descriptive variables of patients receiving an IP monitor or an EVD

	IP monitor (*n* = 739)	EVD (*n* = 139)	*p*-value
Age (median [IQR])	46 [28–61]	48 [27–63]	0.63
Male sex (%)	546 (74)	106 (76)	0.63
Glasgow Coma Scale (median [IQR])	6 [3–10]	5.5 [3–10]	0.53
Glasgow Coma Scale Motor score (median [IQR])	3 [1–5]	2 [1–5]	0.15
Pupillary reactivity at baseline (*N* (%))			0.05
Pupils reactive	530 (75)	80 (64)	
One pupil unreactive	61 (9)	16 (13)	
Both pupils unreactive	116 (16)	28 (23)	
Injury Severity Scale (median [IQR])	34 [25–48]	34 [25–43]	0.99
Cause of injury (%)			0.36
Road traffic accident	345 (49)	59 (43)	
Fall	259 (37)	58 (43)	
Violence/suicide	49 (7)	12 (9)	
Other	52 (7)	7 (5)	
Traumatic subarachnoid hemorrhage (%)	548 (86)	99 (85)	0.99
Presence of an epidural hematoma (%)	137 (21)	27 (23)	0.75
Presence of a subdural hematoma (%)	376 (59)	73 (63)	0.48
Presence of a skull fracture (%)	430 (69)	82 (72)	0.56
Compression of basal cisterns (%)	297 (47)	60 (53)	0.29
Midline shift > 5 mm (%)	196 (31)	37 (32)	0.87
Presence of an intraventricular hematoma (%)	207 (32)	47 (41)	0.11
Predicted prevalence of 6-month mortality (median [IQR])	0.15 [0.06–0.35]	0.26 [0.12–0.51]	**0.001**
Predicted prevalence of 6-month unfavorable outcome (median [IQR])	0.57 [0.36–0.75]	0.67 [0.47–0.81]	0.05

### Baseline characteristics

Patients included were predominantly male (74% in the IP monitor and 76% in the EVD group), with a median age of 46 years in the IP and 48 years in the EVD group (Table 1). Most common trauma mechanisms were road traffic accidents in 48% of cases and falls in 38% of all cases. The median GCS on presentation was 6 in the IP group and 5.5 in the EVD group. The majority of patients had equal and reactive pupils (75% in the IP monitor group and 64% in the EVD group). There were no statistically significant differences in baseline characteristics except for the higher prevalence of unreactive pupils in the EVD group in the subset of patients included in the IV analysis (Supplementary Table 2). In both the complete sample and in the sample for the IV analysis, the predicted mortality was higher in the EVD group (Table 1, Supplementary Table 2). The vast majority of devices were inserted within the first 12 h, with more than one-third being inserted in the first 6 h (Table 2, Supplementary Fig. 3).

**Table 2:** Characteristics of ICU therapy and emergency surgical therapy for the entire sample. *TIL*, therapy intensity level, a composite measure indicating the extent to which various therapies are used to control ICP; *CSF*, cerebrospinal fluid

	IP monitor (*n* = 739)	EVD (*n* = 139)	*p*-value
ICP monitoring inserted within *N* (%)			**0.04**
< 1 h	8 (1)	6 (4)	
1–3 h	45 (6)	7 (5)	
3–6 h	219 (30)	45 (33)	
6–12 h	286 (39)	44 (33)	
> 12 h	173 (24)	33 (24)	
Reason for monitoring ICP *N* (%)			0.49
Guideline criteria	257 (35)	37 (27)	
Radiological signs of raised ICP	192 (26)	40 (29)	
Clinical suspicion raised ICP	215 (29)	45 (32)	
Anesthesia or mechanical ventilation required for extracranial injuries	29 (4)	6 (4)	
To inform surgical indication for mass lesion	23 (3)	7 (5)	
Other	22 (3)	4 (3)	
ICP monitoring characteristics			
Median ICP per day (IQR)*	11 [8–14.5]	12 [9.5–15.5]	**0.01**
Number of instances of ICP > 20 mmHg (median[IQR])	2 [0–7]	2 [0–8]	0.47
Number of instances of ICP > 25 mmHg (median[IQR])	0 [0–2]	0 [0–3]	0.43
Duration of ICP monitoring (days, median[IQR])	6.3 [3.4–10.6]	7.5 [4.3–12.3]	0.07
Number of ICP time points recorded (median [IQR])	67 [35.5–84]	65 [29–86]	0.49
Ratio of ICP > 20 mmHg from all measured time points (median[IQR])	0.03 [0–0.13]	0.03 [0–0.13]	0.36
Ratio of ICP > 25 mmHg from all measured time points (median[IQR])	0 [0–0.03]	0 [0–0.05]	0.36
Therapy intensity level (TIL) and use of third tier therapies			
Median TIL per day (IQR)	5 [3–9]	7 [3.5–11]	0.07
Median TIL per day (without points for drained CSF)	5 [3–9]	5 [2.25–9.25]	0.8
Median CSF drained per day, ml (IQR)	0 [0, 0]	75.5 [9–162.5]	< 0.001
Hypothermia *N* (%)	161 (22)	37 (30)	0.07
Barbiturate coma *N* (%)	259 (35)	56 (45)	0.05
Decompressive craniectomy > 12 h after monitor insertion *N* (%)	54 (7)	12 (9)	0.71
Emergency surgical therapy			
Type of surgery *N* (%)			** < 0.001**
None	416 (57)	57 (43)	
Extra- and intracranial	21 (3)	10 (8)	
Extracranial	91 (12)	11 (8)	
Intracranial	206 (28)	54 (41)	
Type of intracranial surgery: *N* (%)			** < 0.001**
Craniotomy for hematoma/contusion	125 (55)	25 (39)	
Emergency decompressive craniectomy	72 (32)	13 (20)	
Depressed skull fracture	13 (6)	2 (3)	
Other	17 (8)	24 (38)	
Complications			
Meningitis/ventriculitis (%)	31 (4)	9 (7)	0.34
Delayed hematoma (%)	102 (14)	36 (26)	**0.001**
Any complications, including device malfunction *N* (%)	208 (29)	48 (35)	0.16
Cessation of ICP monitoring			
Reason to stop ICP monitoring			0.66
Clinically improved N (%)	133 (21)	17 (20)	
ICP stable and < 20 mmHg N (%)	383 (62)	50 (59)	
Monitor/catheter failure *N* (%)	23 (4)	2 (2)	
Patient considered unsalvageable *N* (%)	40 (6)	6 (7)	
Patient died *N* (%)	22 (3)	6 (7)	
Other *N* (%)	22 (3)	4 (5)	

*TIL*, therapy intensity level

### Choice of device

The significant predictors for use of EVD over IP in the fixed-effects model were one unreactive pupil (OR [95% CI] 1.96 [1.05–3.65]), emergency intracranial surgery (OR [95% CI] 2.44 [1.45–4.09]), and emergency intracranial and extracranial surgery (OR [95% CI] 4.08 [1.71–9.71], and also significant in the random-effects analysis: OR [95% CI] 3.38 [1.03–11.08]) (Supplementary Table 3). The Nagelkerke *R*^2^ of the model with patient characteristics alone was 0.28. The addition of a random intercept for center conditional increased the Nagelkerke *R*^2^ to 0.57 (Supplementary Table 3). Of the EVD patients, 24 received a ventricular device with a mounted pressure sensor; these were excluded from the intention-to-treat analysis and added to the as-treated analysis in the EVD group.

### Therapy and length of stay

**Fig. 1:**
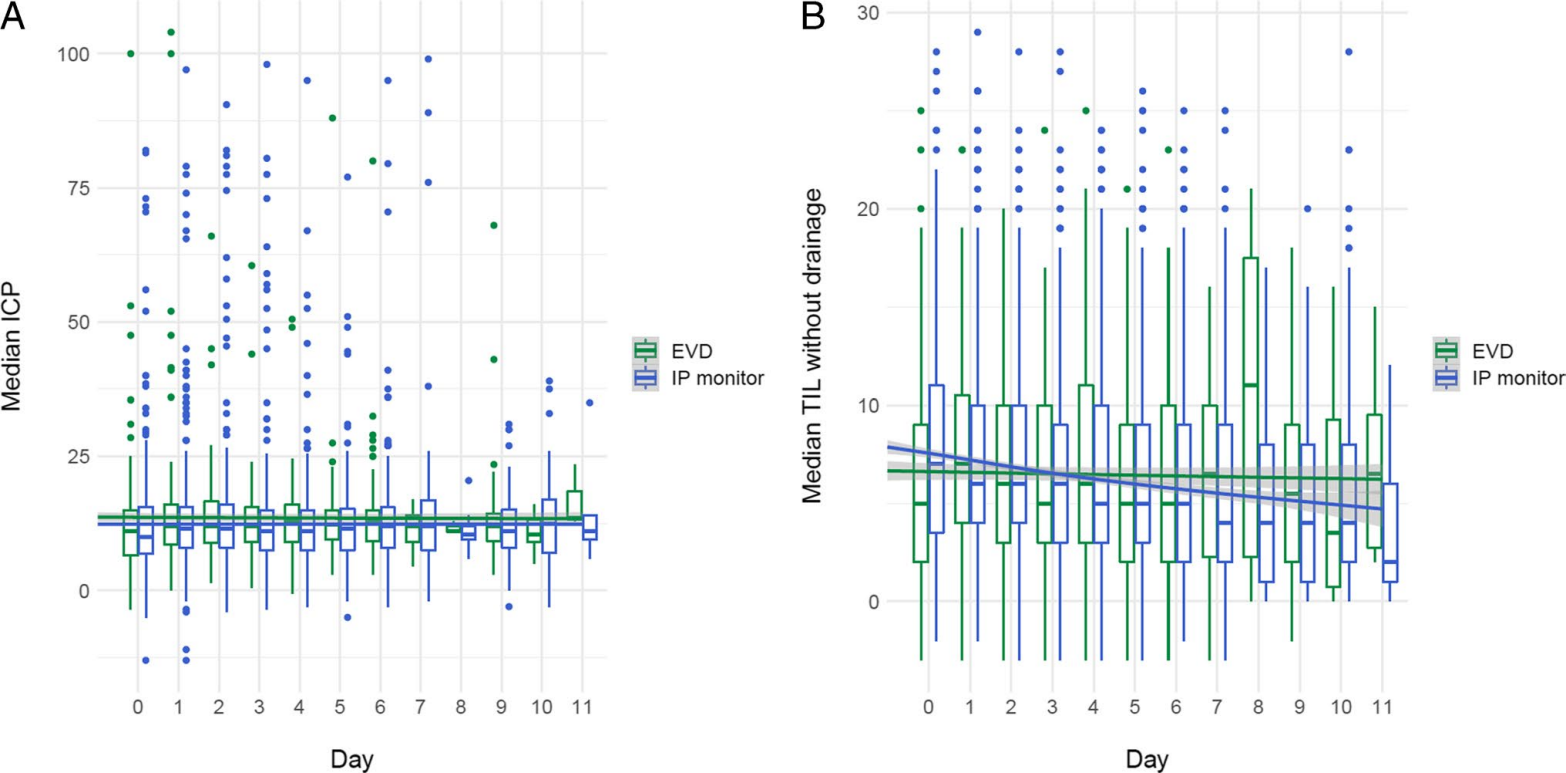
Median ICP and therapy intensity level (TIL) (scores for draining CSF were not taken into consideration) per day for both groups. **A** Median ICP per day for both groups. **B** Median TIL per day for both groups

The median daily ICP was significantly different between the two groups, but the difference was clinically irrelevant, with 11 mmHg in the IP monitor group and 12 mmHg in the EVD group (*p* = 0.01) (Table 2, Fig. 1A). Duration of ICP monitoring was 6.3 days in the IP monitor group (IQR 3.4–10.6) and 7.5 days (IQR 4.3–12.3) in the EVD group. The ratio of high ICP measurements, defined as the number of instances with an ICP measured above 20 or 25 divided by the total number of time points measured, was not different between the two groups (Table 2).

The median daily therapy intensity level (TIL) was not different between the two groups, even when excluding CSF drainage (median [IQR] 5 [3–9] in the IP group and 7 [3.5–11] in the EVD group) (Table 2, Fig. 1B). A median of 75 ml CSF was drained daily in the EVD group (Table 2).

The mean hospital length of stay (HLOS) and ICU length of stay (ICU LOS) were higher in the EVD group (Table 3), with the mean number of days spent in the ICU being 70% higher in the EVD group, rate ratio = 1.7, 95% CI [1.34–2.12], adjusted IV analysis.

**Table 3:** Effect of placing an EVD when compared to placing an IP monitor for ICP-directed management. IV analysis was performed for a sub-sample of centers including more than 10 patients. For outcomes that did not lend themselves to IV analysis, the results of the entire cohort are reported. (OR values above 1 reflect higher rates in the EVD group; bold values denote statistically significant results and the main analysis). *OR*, odds ratio; *95% CI*, confidence interval

Outcome	Unadjusted regression analysis	Multivariable adjustment	IV analysis	**Adjusted IV analysis**
Mortality at 6 months, OR (95% CI)	1.36 (0.86–2.14)	1.12 (0.65–1.91)	1.27 (0.59–2.59)	1.03 (0.40–2.48)
GOS-E at 6 months dichotomized (unfavorable outcome), OR (95% CI)	1.08 (0.71–1.66)	0.92 (0.57–1.49)	0.99 (0.53–1.94)	0.74 (0.36–1.52)
Length of hospital stay*, rate ratio of days (95% CI)	1.15 (0.99–1.35)	1.13 (0.97–1.31)	1.15 (0.89–1.45)	1.14 (0.89–1.42)
Length of ICU stay*, rate ratio of days (95% CI)	1.37 (1.21–1.56)	1.35 (1.19–1.53)	1.72 (1.35–2.16)	**1.70 (1.34–2.12)**
Decompressive craniectomy**, OR (95% CI)	1.11 (0.53–2.35)	1.00 (0.45–2.19)	0.64 (0.10–2.09)	0.68 (0.11–2.45)
Hypothermia use, OR (95% CI)	1.56 (1.02–2.38)	1.54 (0.98–2.42)	NA	NA
Barbiturate coma use, OR (95% CI)	1.48 (0.99–2.20)	1.52 (1.00–2.32)	NA	NA
Use of any third tier therapy (barbiturate coma, hypothermia, decompressive craniectomy) OR (95% CI)	1.35 (0.91–2.01)	1.35 (0.90–2.04)	NA	NA
Overall complications OR (95% CI)	1.33 (0.91–1.95)	1.25 (0.85–1.86)	NA	NA
Complications: Infection OR (95% CI)	1.54 (0.73–3.24)	1.58 (0.74–3.41)	NA	NA
Complications: Delayed hematoma OR (95% CI)	2.15 (1.40–3.32)	**2.04 (1.28–3.24)**	NA	NA
Complications: Device malfunction OR (95% CI)	0.52 (0.27–1.00)	0.53 (0.28–1.02)	NA	NA
Ratio of instances of ICP > 20 mmHg OR (95% CI)	1.22 (0.88–1.68)	1.20 (0.86–1.67)	NA	NA
Ratio of instances of ICP > 25 mmHg OR (95% CI)	1.25 (0.88–1.75)	1.25 (0.88–1.77)	NA	NA
Mortality before discharge OR (95% CI)	1.53 (1.00–2.35)	1.36 (0.85–2.19)	NA	NA
Mortality in the ICU OR (95% CI)	1.56 (1.00–2.43)	1.42 (0.87–2.32)	NA	NA

The multivariable adjustment method used age, motor GCS, pupils, sex, CT variables, and total ISS as potential confounders

^*^Patients who died in hospital/at the ICU were excluded from these analyses to avoid biased estimates (same follow-up for the rest). The rate ratios of these analyses can be interpreted as: “The mean number of days increased by a factor of x for patients in the EVD group”

^**^For this analysis, patients receiving a primary decompressive craniectomy were excluded

The main analysis was considered the adjusted IV analysis for outcomes that lent themselves to this analysis

A total of 187 patients (25%) who were primarily monitored with an IP monitor crossed over and required an EVD later on during ICP treatment due to refractory high ICP.

### Outcomes

**Fig. 2:**
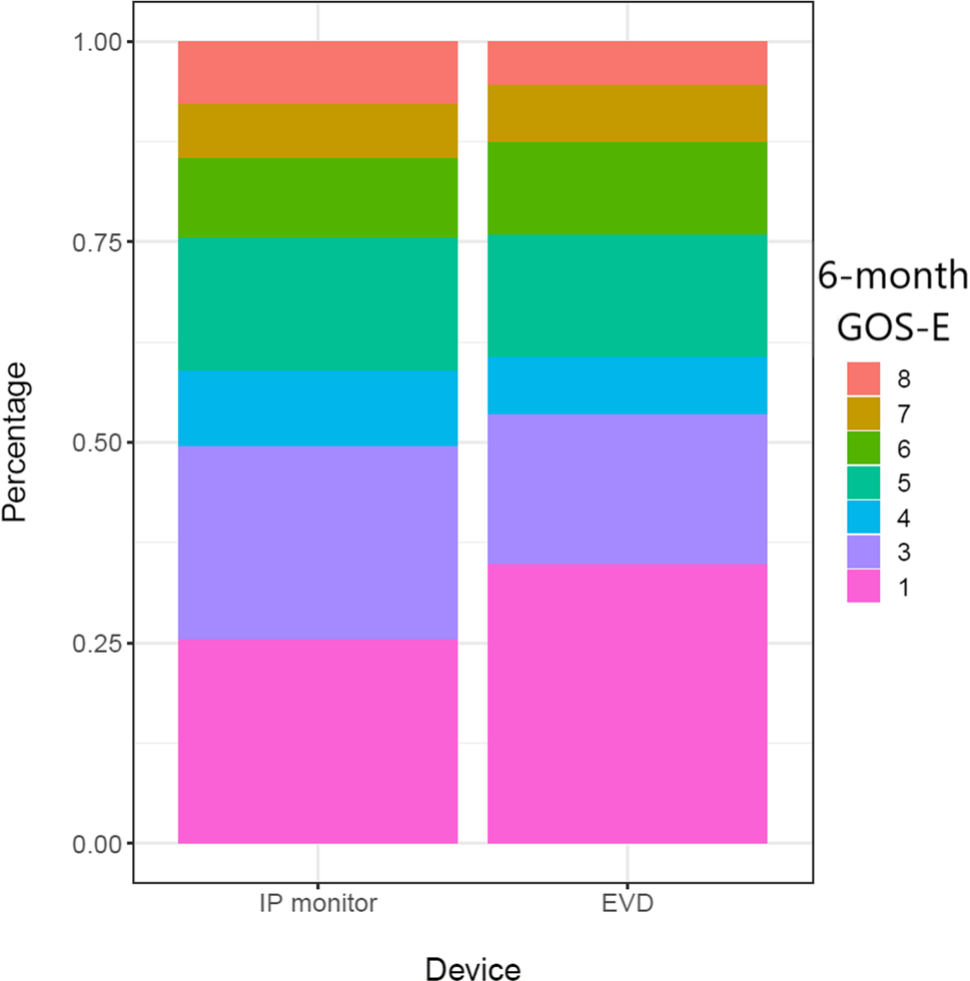
Absolute GOS-E numbers for both groups. Because of the merging of postal questionnaires and in-person interviews, 2 and 3 were merged in one category, represented as 3 below

The 6-month GOSE (dichotomized) did not differ between the two groups, EVD versus IP OR 0.74 and 95% CI [0.36–1.52], adjusted IV analysis (Fig. 2, Table 3). Mortality at 6 months did not differ significantly between the two groups (aOR and 95% CI 1.03 [0.40–2.48]).

The need for decompressive craniectomy was similar between groups, but the analysis was underpowered (aOR and 95% CI 0.68 [95% CI: 0.11–2.45], adjusted IV analysis) (Table 3).

The need for any third-tier therapies was not different between the two groups (aOR 1.35 and 95% CI [0.90–2.04], multivariable regression) (Table 3).

The rate of overall complications was similar between groups (aOR 1.58 and 95% CI [0.74–3.41], multivariable regression). The risk of a delayed hematoma was significantly higher in the EVD group (aOR 2.04 and 95% CI [1.28–3.24]) (Table 3).

Because of the higher risk of delayed hematoma in both the main and sensitivity analyses, we decided to further explore the relationship between the amount of CSF drained and the risk of delayed hematoma using multivariable logistic regression. The analysis showed no association between either daily median drained volume or total drained volume and the risk of developing a delayed hematoma (aOR [95% CI] 1.0 [0.9–1.1] for both covariates). The only variable associated with the risk of developing a delayed hematoma was having bilateral fixed pupils at baseline (aOR [95% CI] 4.2 [1.35–13.69]).

We also explored the relationship between CSF drainage and the need for third tier therapies, adjusting for covariates associated with the need for higher TIL, reported in a previous work [8]. When comparing the IP monitoring group with the “early EVD” (primary intention) group and “late EVD” group (patients who received an EVD after an IP monitor), the late EVD group had a significantly higher need for third tier therapies (aOR [95% CI] 2.26 [1.43–3.4]).

### Sensitivity analyses

The “as-treated” analysis involved moving the 187 patients that received an EVD at a later time point after IP insertion due to refractory high ICPs to the “EVD” group. This left 552 patients in the IP monitor group and 336 in the EVD group. The 336 patients in the EVD group included 10 patients who had both monitors from the beginning and who were excluded from the “intention-to-treat” analysis. At baseline, the EVD group had more subdural hematomas, the basal cisterns were more often compressed, and both the predicted mortality and predicted unfavorable outcome at 6 months were higher.

The EVD group had higher overall TILs, a higher ratio of time points with an ICP > 20 or 25 mmHg and needed overall more third tier therapies. Interestingly, while this change did affect the coefficients, and the EVD group had an overall higher rate of complications (aOR [95% CI] 2.17 [1.61–2.94], multivariable analysis) and a higher need for third tier therapies (aOR [95% CI] 2.22 [1.66–2.97], multivariable analysis), both 6-month mortality and unfavorable functional outcome were similar between groups (mortality aOR [95% CI] 1.55 [0.67–3.49] and unfavorable functional outcome 1.04 [0.52–2.13], adjusted IV analysis) (Supplementary Table 4).

The sensitivity analysis which employed the 12-month GOS-E supported our conclusions, with all results remaining the same, no difference in mortality or unfavorable functional outcome for both the “intention-to-treat” as for the “as-treated” analyses. The level of missing covariate data for clinical baseline variables was between 0 and 6.4% (Supplementary Table 5). For the baseline CT covariates, the level varied between 13.9 and 15.5%. The complete case sensitivity analysis also revealed similar effect size estimates, with no difference in terms of primary outcome (Supplementary Table 6).

## Discussion

Our study found no major differences in functional outcome or mortality when comparing ICP monitoring with EVDs versus IP monitors to guide ICP management. Draining cerebrospinal fluid concomitantly with ICP measurement does not confer benefit in itself for ultimate clinical outcome. The risk of a delayed hematoma was higher and length of stay was prolonged in the patients managed with EVDs, but this did not translate to worse functional outcomes. A quarter of patients initially treated with IP monitoring had to cross over to EVD because of refractory high ICP during their ICP management period.

We performed a CER approach using data from one of the largest prospective TBI cohorts to date to compare the outcomes of the EVD group with those of the IP monitor group. The evidence so far is poor and contradictory [3, 11, 21], making practice variation the rule, not the exception [20]. While this would not be an issue if indeed the two methods are equally effective [21], reports suggesting the superiority of one or the other treatment modalities [3, 11] made further research necessary. In our data, mortality and functional outcomes did not differ between the two groups.

Our meta-analysis of all studies up to 2018 shows no difference in terms of mortality or functional outcome between patients given IP monitors and those given EVDs [21]. The only RCT on this topic [11], including 122 patients, with relatively low risk of bias, showed lower mortality, better functional outcome, and a reduced need for decompressive craniectomy in patients receiving an EVD. A more recent study, using state-of-the-art statistical methods for confounder adjustment [3], showed significantly worse functional and neuropsychological outcomes in patients with EVDs. Of interest, we re-ran the meta-analysis using the new data provided by Bales et al. together with data from the current study. We found no difference, either in terms of mortality or functional outcome. From a methodological standpoint, however, internal validity of included studies should always take precedence when interpreting the results of any meta-analysis, and from this point of view, despite the pooled result, the study by Liu et al. remains the most methodologically sound [11]. The only issue with this RCT is its limited generalizability given the likely unrepresentative sample of 122 patients (relatively young patients, less than 20% being above 60 years of age and 25% of the sample had a GCS above 8).

We had hypothesized a lower therapy intensity level [25] (TIL) in the EVD group given that drainage of CSF has an impact on lowering ICP. Less need for ICP-lowering therapy when ICP is monitored with an EVD has been suggested by previous research [11]. The TIL was not significantly different between the two groups, but the median ICP showed a statistically significant difference. However, this difference was of 1 mmHg, rendering it clinically irrelevant. Furthermore, ICP control was achieved to the same extent in both groups. We hypothesized that the presence of an EVD, actively draining CSF, might prevent the use of additional aggressive ICP-lowering treatments. This, however, was also not sustained by our data showing no differences in the use of third tier therapies between the two groups. One-fourth of patients in the IP monitoring group did eventually cross over and required an EVD for ICP control. The risk of complications, such as infection, has often been quoted as a reason to not use EVDs to monitor and treat TBI patients [21]. In the main analysis, we did not find any difference in terms of infections between the IP and EVD group. In the sensitivity analysis, however, the risk of infection was higher in the EVD group, as expected, but this did not translate into worse clinical outcomes.

For the “average” TBI patient presenting with an indication for monitoring, choosing an EVD instead of an intraparenchymal monitor does not appear to lead to better functional outcome or lower mortality. There is a higher risk of delayed hematoma, but we found no evidence to suggest this complication was directly related to the magnitude of the intervention itself, the amount of drained CSF. The unanswered question still remains if patients that actually develop refractory high ICP will benefit from early drainage. This is a slightly different question and patient selection than our present study. It also requires a better characterization of the phenotype of patients that develop refractory ICP. Our cohort included patients who lent themselves to ICP control with relative ease. In deciding whether to monitor with an IP monitor or an EVD, however, clinicians should take into account the fact that in our cohort, one-fourth of patients crossed over, and required an EVD later on during ICP monitoring for refractory high ICP. The “as treated” EVD group had overall higher TILs and need for third tier therapies. This might be related to confounding by indication present in the data, and is nothing more than an association. We cannot exclude the potential explanation that placing an EVD later on during ICP treatment leads to a higher need for third tier therapies. We were unfortunately unable to model the relationship “time to late EVD,” but this finding suggests clinicians should have a low threshold for placing EVDs early on during ICP treatment.

Both EVDs and IP monitors inform treatment and ICP-lowering therapies based on these monitoring modalities lead to similar outcomes. Our data shows that, when EVDs are used as a first intention, there is no difference in outcome when compared to IP monitors. When starting with an IP monitor, however, a quarter of patients do require this step (EVDs) in their ICP management protocol. We feel this very high cross-over rate should raise awareness to the potential use of EVDs, even though on a group level they do not improve the primary outcomes. Further, if both approaches (IP and EVD) are equally effective in terms of outcome, costs become an issue. We note that EVDs are considerably cheaper than IP devices.

### Limitations

Despite CENTER-TBI including a generous cohort of TBI patients, we were only able to include 136 patients with EVDs in the main analysis. In the start-up phase of our study, we conducted “provider profiling” of participating centers. Within these self-administered questionnaires, we profiled the “standard of care” across participating centers. Whereas 60% of centers indicated using both modalities and 8% indicated using only EVDs for monitoring [5], we could not confirm this in the core data of the patients included. We used IV analysis for our primary outcomes using center as a moderate strength instrument, but despite explaining a considerable amount of variation, there likely remains a significant amount of residual confounding. For most patients in our study, most of the measured ICP values were under 15, indicating that both groups of patients mostly had controlled ICP. ICP was also not measured using the “high resolution” package [2], which might further confound results, as “instantaneous” measurements were not available and the influence of short bursts of high ICP could not be evaluated.

An issue not dealt with in our study is whether CSF should be drained continuously or intermittently. The current guidelines recommend continuous drainage, but this recommendation is based on two observational studies which include a small number of patients, leaving this question still open for debate [4, 18].

We recognize the limitations of our study and of its interpretation. If anything, the study illustrates the challenges and complexity of a CER design within an observational study in the specific field of TBI, and as such may serve to stimulate debate and reflection on the use of more advanced methodologies for future research.

## Conclusion

We found no major differences in clinical outcomes of patients undergoing IP monitor- or EVD-based ICP treatment, using a comparative effectiveness design. A quarter of patients who received an IP monitor as a first intention eventually required an EVD. The prevalence of complications was higher in the EVD group.

## Supplementary Information

Below is the link to the electronic supplementary material.

Supplementary file1 (DOCX 213 KB)
