# Supplementary material for: Comparative effectiveness of intracranial hypertension management guided by ventricular versus intraparenchymal pressure monitoring: a CENTER-TBI study
Source: Acta Neurochir (Wien). 2022 Jun 1;164(7):1693–705. doi: 10.1007/s00701-022-05257-z (PMC9233652; doi:10.1007/s00701-022-05257-z)
Supplement: Supplementary file 1 — Supplementary file1 (DOCX 213 KB) [file 701_2022_5257_MOESM1_ESM.docx]

Supplementary Appendix

**Tables**

Table 1, The primary and secondary outcomes of this study

| **Outcome** | |
| --- | --- |
| **Co-Primary** | 6-month mortality |
|  | 6-month unfavorable functional outcome measured by the Glasgow Outcome Scale-Extended (GOSE), defined as GOS-E<5 |
|  | |
| **Secondary**  *ICU treatment variables* | Mortality in the ICU  Mortality at hospital discharge  Prevalence of cross-over (IP monitor patients needing EVD)  Therapy Intensity Level (TIL), with and without scores for CSF drainage |
|  | Daily median ICP |
|  | Ratio of time points with ICP above 20 mmHg and above 25 mmHg out of all recorded ICP time points |
|  | Length of ICU stay (LOICUS) |
|  | Length of Hospital stay (LOHS) |
| *Third tier therapies* | Secondary decompressive craniectomy (>12 hours from ICP monitor insertion) |
|  | Use of barbiturates |
|  | Use of hypothermia |
|  | Use of any third-tier therapy |
| *Complications* | Delayed hematoma |
|  | Meningitis/Ventriculitis |
|  | Device malfunction |
|  | Any complication |

Table 2, Baseline descriptive variables of patients included in the instrumental variable analysis receiving IP monitors or EVD, in a sub-sample of centers that included more than 10 patients

|  | IP monitor (n=639) | EVD (n=115) | | p-value |
| --- | --- | --- | --- | --- |
| Age (median [IQR]) | 46 [27 - 61] | 46 [26.5 - 61] | | 0.89 |
| Male sex (%) | 473 (74) | 87 (75) | | 0.80 |
| Glasgow Coma Scale (median [IQR]) | 6 [3 - 10] | 5.5 [3 - 10] | | 0.49 |
| Glasgow Coma Scale Motor score (median [IQR]) | 2.5 [1 - 5] | 1 [1 - 5] | | 0.11 |
| Pupillary reactivity at baseline (N (%)) |  |  | | **0.006** |
| Pupils reactive | 456 (74) | 63 (59) | |  |
| One pupil unreactive | 54 (9) | 16 (15) | |  |
| Both pupils unreactive | 107 (17) | 28 (26) | |  |
| Injury Severity Scale (median [IQR]) | 34 [25 - 48] | 35 [25.5 - 50] | | 0.37 |
| Cause of injury (%) |  |  | | 0.86 |
| Road Traffic Accident | 300 (49) | 55 (48) | |  |
| Fall | 221 (36) | 44 (39) | |  |
| Violence/suicide | 45 (7) | 9 (8) | |  |
| Other | 44 (7) | 6 (5) | |  |
|  |  |  | |  |
| Traumatic subarachnoid hemorrhage (%) | 490 (86) | 88 (85) | | 0.97 |
| Presence of an epidural hematoma (%) | 116 (20) | 23 (22) | | 0.75 |
| Presence of a subdural hematoma (%) | 338 (59) | 67 (65) | | 0.33 |
| Presence of a skull fracture (%) | 383 (69) | 72 (71) | | 0.68 |
| Compression of basal cisterns (%) | 258 (46) | 52 (52) | | 0.31 |
| Midline shift > 5 mm (%) | 172 (30) | 33 (32) | | 0.79 |
| Presence of an intraventricular hematoma (%) | 184 (32) | 40 (39) | | 0.24 |
| Predicted prevalence of 6-month mortality (median [IQR]) | 0.16 [0.06 - 0.35] | 0.23 [0.12 - 0.50] | | **0.004** |
| Predicted prevalence of 6-month unfavorable outcome (median[IQR]) | 0.57 [0.36 - 0.75] | 0.67 [0.47 - 0.80] | | 0.07 |
| *Predicted based on all variables in this table, using logistic regression. | | |  |  |

Table 3, Logistic regression analysis of variables associated with the choice to monitor with an EVD versus IP as the reference and the Nagelkerke R^2^ of the prediction model. On the right, predictors of the use of an EVD with IP monitor as reference with center added as a random effect. Results in bold are statistically significant.

|  | Model without center | Model with center as random effect |
| --- | --- | --- |
| Two reactive pupils | Ref | Ref |
| One pupil unreactive | **1.96 (1.05 - 3.65)** | 1.54 (0.64 - 3.68) |
| Both pupils unreactive | 1.60 (0.97 - 2.64) | 1.86 (0.93 - 3.72) |
| Emergency intracranial surgery | **2.44 (1.45 - 4.09)** | 2.08 (0.98 - 4.41) |
| Emergency extracranial surgery | 0.78 (0.36 - 1.71) | 0.73 (0.29 - 1.88) |
| Emergency intracranial and extracranial surgery | **4.08 (1.71 - 9.71)** | **3.38 (1.03 - 11.08)** |
| Presence of midline shift | 0.65 (0.39 - 1.07) | 0.59 (0.28 - 1.25) |
| Intraventricular hemorrhage | 1.35 (0.86 - 2.12) | 1.52 (0.84 - 2.77) |
|  |  |  |
| Nagelkerke R^2^ | 0.28 | 0.57 |

Table 4 – Sensitivity analysis “as-treated”, the equivalent of Table 3 in the main text. Patients with an EVD at any time point are included in the EVD group. Effect of placing an EVD when compared to placing an IP monitor for ICP-directed management. IV analysis was performed for a sub-sample of centers including more than 10 patients. For outcomes that did not lend themselves to IV analysis, the results of the entire cohort are reported. Results in bold are statistically significant. (OR values above 1 reflect higher rates in the EVD group). OR= odds ratio, 95% CI= Confidence Interval

| Outcome | Unadjusted regression analysis | Multivariable adjustment | IV analysis | **Adjusted IV analysis** |
| --- | --- | --- | --- | --- |
| Mortality at 6 months, OR (95% CI) | 1.46 (1.05 - 2.04) | 1.41 (0.96 - 2.08) | 1.55 (0.76 - 3.01) | 1.55 (0.67 - 3.49) |
| GOS- E at 6 months dichotomized (unfavorable outcome), OR (95% CI) | 1.31 (0.94 - 1.84) | 1.32 (0.92 - 1.91) | 1.10 (0.59 - 2.10) | 1.04 (0.52 - 2.13) |
| Length of hospital stay*, rate ratio of days (95% CI) | 0.73 (0.55 - 0.96) | 0.75 (0.56 - 0.99) | 0.87 (0.47 - 1.55) | 0.88 (0.50 - 1.57) |
| Length of ICU stay*, rate ratio of days (95% CI) | 1.26 (1.13 - 1.41) | 1.22 (1.10 - 1.37) | 1.04 (0.79 - 1.34) | 1.06 (0.81 - 1.34) |
| Decompressive craniectomy**, OR (95% CI) | 1.46 (1.33 - 1.61) | 1.44 (1.31 - 1.58) | 1.50 (1.19 - 1.86) | **1.54 (1.26 - 1.90)** |
| Hypothermia use, OR (95% CI) | 2.20 (1.60 - 3.03) | **2.16 (1.54 - 3.03)** | NA | NA |
| Barbiturate coma use, OR (95% CI) | 2.03 (1.53 - 2.69) | **2.10 (1.56 - 2.81)** | NA | NA |
| Use of any third tier therapy (barbiturate coma, hypothermia, decompressive craniectomy) OR (95% CI) | 2.19 (1.66 - 2.90) | **2.22 (1.66 - 2.97)** | NA | NA |
| Overall complications OR (95% CI) | 2.29 (1.70 - 3.07) | **2.17 (1.61 - 2.94)** | NA | NA |
| Complications: Infection OR (95% CI) | 4.84 (2.43 - 9.64) | **4.85 (2.37 - 9.89)** | NA | NA |
| Complications: Delayed hematoma OR (95% CI) | 3.59 (2.45 - 5.25) | **3.52 (2.38 - 5.21)** | NA | NA |
| Complications: Device malfunction OR (95% CI) | 0.96 (0.64 - 1.44) | 0.99 (0.66 - 1.48) | NA | NA |
| Ratio of instances of ICP> 20 mmHg  OR (95% CI) | 1.74 (1.36 - 2.23) | **1.66 (1.29 - 2.13)** | NA | NA |
| Ratio of instances of ICP> 25 mmHg  OR (95% CI) | 1.66 (1.28 - 2.16) | **1.61 (1.23 - 2.10)** | NA | NA |
| Mortality before discharge OR (95% CI) | 1.45 (1.03 - 2.03) | 1.41 (0.97 - 2.06) | NA | NA |
| Mortality in the ICU OR (95% CI) | 1.38 (0.97 - 1.97) | 1.33 (0.90 - 1.96) | NA | NA |
| The multivariable adjustment method used age, motor GCS, pupils, sex, CT variables and total ISS as potential confounders.  *Patients who died in hospital/at the ICU were excluded from these analyses to avoid biased estimates (same follow-up for the rest). The rate ratios of these analyses can be interpreted as: “The mean number of days increased by a factor of x for patients in the EVD group”.  **For this analysis, patients receiving a primary decompressive craniectomy were excluded.  The main analysis was considered the adjusted IV analysis for outcomes that lent themselves to this analysis. | | | | |

Table 5 – Percentage of missing data per variable

|  | **Missing (%)** |
| --- | --- |
| Age (median [IQR]) | 0 |
| Male sex (%) | 0 |
| Glasgow Coma Scale (median [IQR]) | 6.4 |
| Glasgow Coma Scale Motor score (median [IQR]) | 2.7 |
| Pupillary reactivity at baseline (N (%)) | 5.6 |
| Pupils reactive |  |
| One pupil unreactive |  |
| Both pupils unreactive |  |
| Injury Severity Scale (median [IQR]) | 0.2 |
| Cause of injury (%) | 4.3 |
| Road Traffic Accident |  |
| Fall |  |
| Violence/suicide |  |
| Other |  |
| Traumatic subarachnoid hemorrhage(%) | 14 |
| Presence of an epidural hematoma (%) | 14 |
| Presence of a subdural hematoma (%) | 14 |
| Presence of a skull fracture (%) | 15.5 |
| Compression of basal cisterns (%) | 14.5 |
| Midline shift > 5 mm (%) | 13.9 |
| Presence of an intraventricular hematoma (%) | 14.1 |

Table 6 – Sensitivity analysis complete cases, “intention-to-treat” analysis. The results inform the effect of placing an EVD when compared to placing an IP monitor for ICP-directed management. IV analysis was performed for a sub-sample of centers including more than 10 patients. (OR values above 1 reflect higher rates in the EVD group). OR= odds ratio, 95% CI= Confidence Interval, IV= instrumental variable

| Outcome | Adjusted IV analysis |
| --- | --- |
| Mortality at 6 months, OR (95% CI) | 0.96 (0.36 - 2.42) |
| GOS- E at 6 months dichotomized (unfavorable outcome), OR (95% CI) | 0.68 (0.29 - 1.50) |
| Length of hospital stay*, rate ratio of days (95% CI) | 1.26 (0.66 - 2.35) |
| Length of ICU stay*, rate ratio of days (95% CI) | 0.97 (0.33 - 2.47) |
| Decompressive craniectomy**, OR (95% CI) | 1.23 (0.54 - 2.86) |

**Figures**

Figure 1, Number of patients per country (above) and center (below), all patients included.

Figure 2, Number of patients per country (above) and per center (below) for centers including more than 10 patients.

| **** |  |
| --- | --- |

Figure 3, Bar plot showing the number of insertions of monitoring devices per hour.
